# Integrating Human and Wildlife Dynamics in Co‐Occurrence Modelling

**DOI:** 10.1002/ece3.70984

**Published:** 2025-02-17

**Authors:** F. Rolle, M. V. Boiani, L. Fardone, F. Gaydou, M. Macario, F. Parentela, V. Ruco, D. Sigaudo, F. Marucco

**Affiliations:** ^1^ Department of Life Sciences and Systems Biology University of Torino Torino Italy; ^2^ Department of Biological Sciences, Conservation Biology Research Group University of Chester Chester UK; ^3^ Ente di Gestione delle Aree Protette delle Alpi Marittime (APAM) Valdieri Italy

**Keywords:** camera traps, detectability, hunting pressure, predator–prey co‐occurrence

## Abstract

In shared environments, where different species interact depending on overlapping resources, complex interspecific interactions emerge, with human activities impacting these dynamics and influencing wildlife abundance and distribution. In the Alps, the presence of multiple species of ungulates, such as roe deer and red deer, and a predator, the wolf, creates a web of spatial and behavioral interactions in an area where farming, hunting and tourism have persisted over time, with tourism recently experiencing a substantial growth. Accounting for these multiple interactions, we modelled the co‐occurrence probabilities of roe deer, red deer and wolves in an area of the Maritime Alps using data derived from 60 camera traps. We applied multi‐species occupancy models to investigate (i) the role of species co‐occurrences in explaining the occupancy of model species across the landscape, (ii) the role of human presence and activities on species occupancy and (iii) the potential effect of the hunting season on the species detection probabilities. Among the identified species, roe deer reported the highest frequency of recorded events and were the most widespread species. We provided important evidence of interspecific dependence, revealing that pairwise interactions among species had a greater impact than only considering individual environmental effects. We documented that the setting of cameras on trails increased the likelihood of detecting wolves but decreased the likelihood of detecting ungulates. Most importantly, the hunting season significantly reduced the likelihood of capturing roe deer, while having no effect on either red deer or wolves. Our results confirmed the relevance of including prey, predators, and human dynamics as a whole. Since the sharing of habitat makes human activities significantly important in defining predator–prey mechanisms, our insights are particularly relevant for defining solutions to optimize human‐wildlife coexistence, especially in a highly anthropogenic system such as Europe.

## Graphical abstract

The article explores interspecific interactions in the Maritime Alps, focusing on roe deer, red deer, wolves and humans. Using data from 60 camera traps, we modelled co‐occurrence probabilities, providing important evidence of interspecific dependence and revealing that pairwise interactions among species had a greater impact than only considering individual environmental effects. Key findings also include the significant impact of hunting on roe deer detection, providing valuable insights for conservation practices in shared environments.

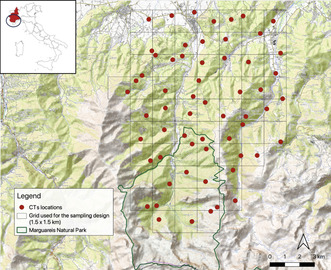

## 1 Introduction

Different species living in shared environments rely on similar resources, leading to complex interspecific interactions that might influence their abundance and distribution (Kavčić et al. 2021). These interactions involve competitive and predator–prey dynamics, making it challenging to understand the factors that enable coexistence, especially in anthropized environments where human activities can further complicate the relationship between species (Van Scoyoc et al. 2023).

Competitive interactions lead to diverse behavioural adaptations (e.g., spatial and temporal avoidance; Kitchen et al. 1999), which are shaped by multiple factors such as resource availability, animal density and degree of overlap between competing species (Kavčić et al. 2021). For instance, in Alpine environments, the co‐occurrence of red deer ( *Cervus elaphus* ) and roe deer ( *Capreolus capreolus* ) has been documented to negatively impact the latter due to a dietary overlap (Borkowski et al. 2021). Previous studies showed that roe deer tend to adapt to competition by exhibiting temporal or spatial avoidance and use of suboptimal resources (Torres et al. 2012), typical adaptations of inferior competitors in herbivore communities (Kavčić et al. 2021).

In addition, sympatric prey species may be exposed to predation risks, which represent another variable in determining direct and indirect behavioural adaptations (Hebblewhite et al. 2005). Predators' presence can indeed alter the community structure and its internal dynamics, or it may also indirectly influence the behaviour of prey, their activity patterns, habitat selection and distribution (Preisser et al. 2005). These non‐lethal effects of predation can often be spatially and temporally structured, creating a heterogeneous landscape of fear that shapes animal behaviour and habitat use patterns (Suraci et al. 2019). Coexistence is facilitated when the shared habitat presents heterogeneity of resources, thus allowing species to partition in different spatial niches (Kavčić et al. 2021).

In this complex system, the growing human footprint is placing significant pressure on wildlife populations worldwide (Bonnot et al. 2013). In landscapes where human activities have transformed the natural environment, there has been a significant increase in spatial diversity within habitats, such as fragmentation of forested areas. Moreover, anthropic disturbances may be perceived by wildlife as equivalent to predation pressures (Suraci et al. 2019). Therefore, when studying species coexistence within complex communities in a highly anthropogenic system such as Europe, it is crucial to acknowledge multiple interspecific interactions with a holistic approach. This aligns with the advancement of statistical models, such as multi‐species occupancy models (MSOMs), which allow for the estimation of the likelihood of multiple species co‐existing, while considering their potential interactions (MacKenzie et al. 2004). Through repeated sampling in space or time, MSOMs can yield unbiased estimates of occupancy probabilities, while adjusting for imperfect detection, thereby providing valuable insights into species co‐occurrence and spatial associations, as well as the factors that influence these patterns (Rota et al. 2016).

The Western Alps represent an ideal scenario to study the complexity of wildlife interspecific interactions in a human‐dominated landscape. The presence of multiple species of ungulates, such as roe deer and red deer, along with the only large predator in the area, the wolf ( *Canis lupus* ), creates a web of spatial and behavioral interactions that act in a highly anthropized system. This region currently presents the highest density of wolf packs of the entire Alps, after being naturally recolonized by the predator since 1996 (Marucco et al. 2023). Consequently, wolves have been present in the Western Alps for almost three decades, providing ample time for humans and prey species to adapt to their presence and consolidate new relationships.

In recent years, camera traps (CTs) have gained considerable recognition as an effective tool to monitor animal presence (Gilbert et al. 2021). In particular, CTs offer an effective approach to simultaneously study different species by avoiding the need to conduct separate sampling campaigns, thereby facilitating the transition from single‐species to multi‐species studies (Iannarilli et al. 2021), other than major advantages of covering large areas and continuously collecting data 24/7.

Considering the benefits afforded by CTs in monitoring wildlife populations and the recent modelling developments, the current study was conducted to examine patterns of co‐occurrence between wild ungulates and wolves in the Western Italian Alps, where coexistence with consolidated human activities represents a crucial factor. In order to understand the complex interplay of competition, predation and effects of human activities, we investigated: (i) the role of species co‐occurrences in explaining the occupancy of model species across the landscape; (ii) the role of human presence and activities as factors that could potentially modify the species occupancy; (iii) the potential effect of the hunting season on the species detection probabilities, with a focus on roe deer, the most important hunted species in the area.

## 2 Materials and Methods

### 2.1 Study Area

**FIGURE 1:**
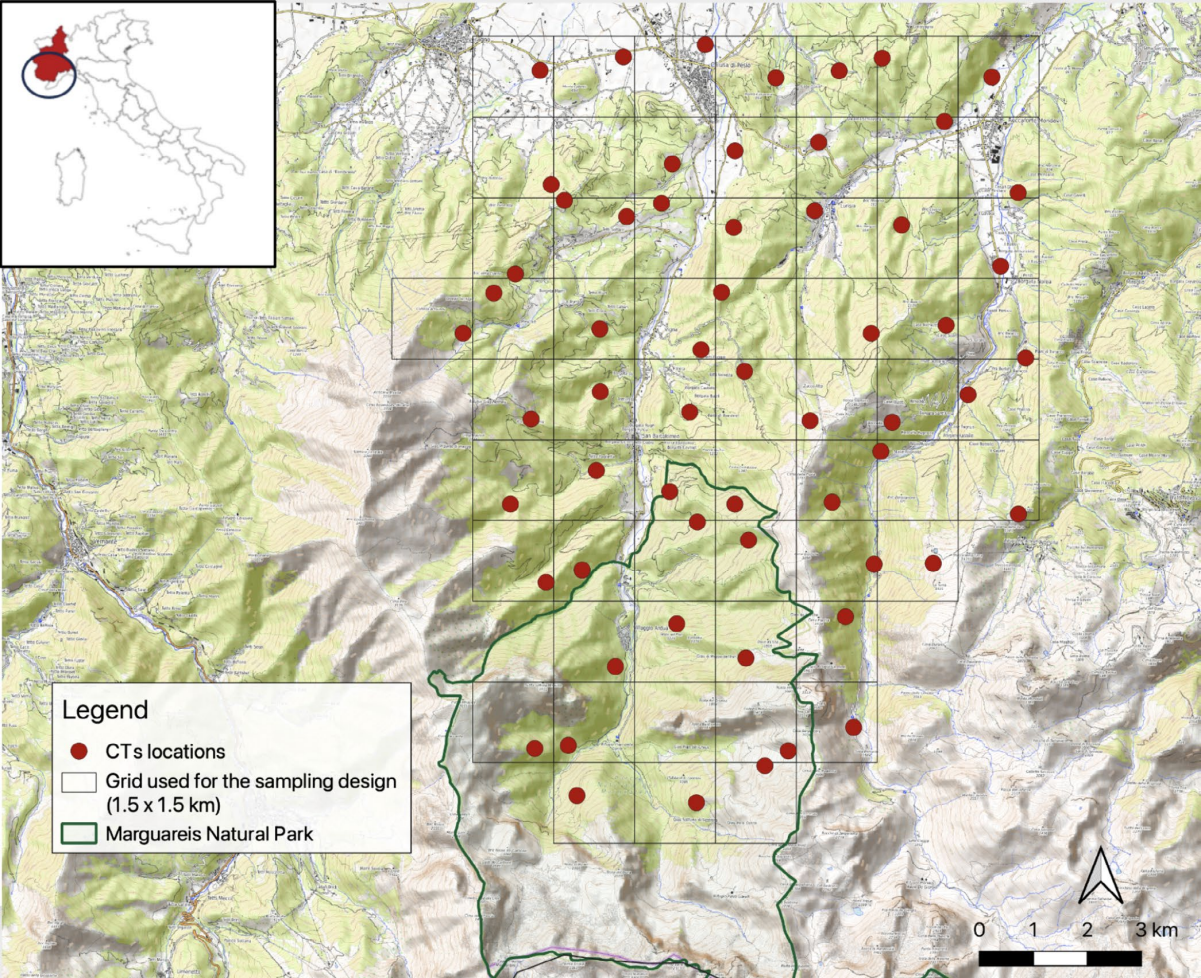
Study area in North‐Western Italy (Piedmont region, in red). The 60‐camera trap (CT) locations are represented with red dots. The black grid illustrates how the study area was divided. Each cell is 1.5 km^2^ and presents one CT in a randomly chosen location.

The camera trap survey was conducted from November 2021 to April 2022 in an area of approximately 136 km^2^ within the Maritime Alps, in the North‐Western part of Italy (Figure 1), where the highest wolf density has been recently documented (Marucco et al. 2023). In Alpine areas with abundant ungulate populations, wolves primarily prey on cervids, with red deer and roe deer being key prey species in the Western Alps (Gazzola et al. 2005, 2007) and in our study area, as documented by Marucco et al. (2008). Red deer, reintroduced to the study area in 1996, are now highly localized within specific zones, rather than showing a widespread scattered distribution. In areas where cervids co‐occur, red deer apply a major pressure on vegetation due to their larger dimensions, impacting roe deer's forage availability (Borkowski et al. 2021), especially in winter (Richard et al. 2010). The area encompasses diverse environments from lowlands to mountains, ranging from 550 m to 2300 m. The study area is partially included inside the Marguareis Natural Park (i.e., almost 30 km^2^ of the total area), where hunting is prohibited and vehicle access restricted. Outside the park, hunting is regulated. The average resident population counts around 30.8 inhabitants/km^2^ concentrated along the valley floors and tourism is promoted by a dense network of waymarked trails.

### 2.2 Sampling Design: CT Deployment

We implemented a standardised protocol aimed at ensuring a uniform distribution of the cameras across the whole study area. The study area was divided into a grid of 60 cells of 1.5 km^2^ each on QGIS 3.16.4 (QGIS 2020). A point was randomly identified inside each cell, thus providing a set of 60 random points to locate the cameras. Access to the initially identified random point was sometimes difficult due to the challenging terrain characteristics. To address this issue, two additional sets of random points were generated as alternative options, prioritising the first random point within a 30‐m tolerance. All cameras were placed on trees, with north‐facing exposure and height from ground ranging between 130 cm (to prevent snow from covering the camera lens) and 170 cm. The cameras were set to take a series of three photographs when triggered, with an interval between trigger events of 0.1 s (i.e., the lowest time available). Following the random positioning process, 11 cameras were located along trails or roads. The models of the cameras were either Keepguard‐KW696 (*n* = 58) or IR‐PLUS‐HD2‐UV595 (*n* = 2). No bait was used for attracting animals. We checked the cameras once a month.

### 2.3 Picture Processing

The images obtained from the CTs were analysed using the dedicated open‐access software Timelapse Image Analyser (Greenberg et al. 2019). The following information was recorded for each image and exported for further analysis: (i) date and time, (ii) CT number, (iii) animal species present in the picture, (iv) total number of individuals, (v) number of new events. Photos of the same occasion have been grouped in a single event, defined by consecutive photos taken within the same moment and activity, close in time, as a single occurrence, as described in other studies (O'Brien et al. 2003; Rød‐Eriksen et al. 2022) to avoid pseudo‐replication and guarantee independence of events. In particular, the first image reporting an animal entering the CT's field of view was counted as one new event, and all subsequent images (such as when the animal grazes without leaving the field of view) were considered as the same event. When we could not surely identify the same individual, we used a 5‐min interval to define a new event, following previous studies (Donini et al. 2025; Henrich et al. 2022). If a photograph included individuals from two different species, it was duplicated to ensure that data from each species could be recorded separately. The category ‘human’ includes both pedestrians/bikers and vehicles.

### 2.4 Data Analysis

We selected an occupancy modelling framework due to its main strength of accounting for imperfect detection (i.e., species present at a site but not detected; MacKenzie et al. 2002). We used Multi‐Species Occupancy Models (MSOMs) that, in addition to Single Species Occupancy Models, integrate interspecific correlations to environmental variables to accurately estimate the factors that affect occupancy.

MSOMs employ three levels of hierarchical modeling frameworks with *j = 1*, …, *J* sites and k = 1, …, *Kj* replicates:

(i) the occupancy model, describing the true presence or absence of a species at each site *J*, which is denoted as:

$$\mathrm{zj}\sim B\text{ernoulli}\left(\mathrm{\psi j}\right).$$

The occupancy probability ψj is modelled using the logistic regression with:

$$\text{logit}\left(\mathrm{\psi j}\right)=\beta 1+\beta 2\cdot X2,j+\ldots +\mathrm{\beta r}\cdot \mathrm{Xr},j$$

that allows for the inclusion of site‐level covariates *X*, whose effects are described by a vector of regression coefficients β.

(ii) the detection model, describing the observed data, modeled as:

$$\mathrm{yj},k\sim \text{Bernoulli}\left(\mathrm{pj},k\cdot \mathrm{zj}\right)$$

where *y*_*j,k*_ represents the detection/non‐detection of the species at site *j* during the *k* replicate survey. Like the occupancy model, the detection model assumes that the data arise from a Bernoulli distribution, but it is conditional on the species being present at the site, as indicated by *z*_*j*_. The detection probability:

$$\text{logit}\left(\mathrm{pj},k\right)=\alpha 1+\alpha 2\cdot V2,j,k+\ldots +\mathrm{\alpha r}\cdot \mathrm{Vr},j,k$$

varies both at the site and survey level, being influenced by a matrix of covariates *V*, whose effects are described by a vector of regression coefficients α.

(iii) the detection process of individual species *i* at site *j* over *K* sampling occasions, which is modeled through a detection frequency variable *y*_*ij*_ and a detection probability *p*_*ij*_ for each sampling occasion *k*:

$$\text{yijk}\sim \text{Binomial}\left(K,\text{pijzij}\right)\;\text{or yijk}\sim \text{Bernoulli}\left(\text{pijkzij}\right).$$

*y*_*ij*_ indicates the detection frequency, and *y*_*ijk*_ is the detection/non‐detection at the *k* ^th^ sampling occasion.

A MSOM is based on the key concept of incorporating multiple matrix layers into the model structure. Instead of using a single detection‐non‐detection matrix, each individual species presents its own matrix, which is then represented through superimposed matrix layers (Rota et al. 2016). Therefore, information from multiple species is combined to estimate the individual species' responses to environmental variables while simultaneously accounting for imperfect detection. We employed the MSOM described by Rota et al. (2016) due to its capability to simultaneously model occupancy dynamics for multiple species, avoiding assuming asymmetric interactions and providing conditions for interspecific independence without requiring additional parameters. Data processing and analysis were conducted in the software R 4.2.2 (R Core Team 2022), using the package *unmarked* (Fiske and Chandler 2011).

### 2.5 Covariate Selection

The occupancy (*p*) and detection (ψ) variables for this study were selected to test specific hypotheses based on literature (Table 1). Because all sites were randomly determined and variably distant from each other, the values used for the analysis were measured within a 200 m radius circular buffer surrounding the CTs. This buffer was designed to represent the habitat conditions recorded by each CT. The covariates in the occupancy model include: (i) measures of human disturbance and (ii) environmental variation. (i) We downloaded road network and settlements digital maps from the online *Geoportale* catalogue (Regione Piemonte 2015), used as indicators of major disturbance to the species. The average daily number of people at each CT trapping site was obtained from CT data directly. (ii) Percentages of open areas and forest cover were obtained from the *Land Cover Piemonte* project data (Regione Piemonte 2021). Terrain ruggedness index (TRI) was calculated through the Digital Elevation Model of Piedmont Region (scale 1:10,000; Regione Piemonte 2008) and used as a proxy for terrain complexity. The spatial data were elaborated in QGIS 3.16.4 (QGIS 2020).

**TABLE 1:** Description of covariates and their expected effect (+, positive or −, negative) on the occupancy (ψ) and detection (*p*) parameters of roe deer (Roe), red deer (Red), wolf and their co‐occurrences.

Parameter	Covariate	Abbreviation	Expected effect single species			Expected effect co‐occurrence			Reference
			Roe deer	Red deer	Wolf	Roe/Red	Roe/Wolf	Red/Wolf	
Detection (*p*)	Visual clarity	Visual	+	+	+				Rota et al. 2016
	On/off trail	Trail	−	−	+				Dickie et al. 2017
	Human presence/absence	Human	−	−	−				Oberosler et al. 2017
	Hunting days	Hunting	−						Bonnot et al. 2013
	Terrain Ruggedness Index	TRI	−	−	−				Wevers et al. 2021
Occupancy (ψ)	Terrain Ruggedness Index	TRI	−	−	+				Wevers et al. 2021
	Forest cover	Forest	+				+		Lovari et al. 2017
	Open areas	Open				+			Kuijper et al. 2009
	Distance to roads	Roads	+			+			Coulon et al. 2008; Bonnot et al. 2013; Petridou et al. 2023; Putzu et al. 2014
	Distance to buildings	Buildings		−			+	−	McDowell et al. 2020
	Number of people per day	Hum_passage			−				Dickie et al. 2017

The covariates in the detection model include: (i) visual clarity index, measured using a 42 × 60 cm chequered board, positioned at 6 points within the CT's viewing range. The visible squares were counted at three angles—central, far right and far left—at both ground level and 130 cm height, at a distance of 18 m from each CT (i.e., maximum range of the sensor of the inferior quality CT). The results were converted to percentages, reflecting visibility, with 100% representing clear visibility (e.g., open field), and lower percentages indicating obstructions like trees/bushes. (ii) Type of trail (i.e., forest road, hiking trail, animal path and off trail), obtained from measurement directly undertaken at the CT site, (iii) TRI, (iv) the record of presence/absence of humans each day, obtained directly from each CT and (v) hunting days, available as open access data (Hunting District CN5, 2022).

All the variables were standardized to an average of 0 and a standard deviation of 1 to attain a comparable range and/or variance (Zipkin et al. 2010). Moreover, variables were checked for any correlation with the *VIF* function of the package *usdm* (Naimi et al. 2014). In cases of correlation detection between two variables, only one of those was included in the model, with a correlation threshold of 0.6 being applied (Dormann et al. 2013).

### 2.6 Statistical Analysis: Set of Models

For each species, we built detection/non‐detection matrices where each CT station represented a sampling site and the time intervals for each repeated survey were grouped as 1 day. We calculated a capture rate by dividing the number of events by the total trap days per hundred (Rovero et al. 2014). We also estimated the naive occupancy as the probability of a species occupying a site, based only on the proportion of sites where it was detected. Afterwards, the modelling framework was divided into three groups of hypothesis testing, which followed the three initial objectives of the study (Table 2), following Rota et al. (2016). A set of candidate models was applied for each objective to test the hypothesis and avoid data dredging (Burnham and Anderson 2002).

**TABLE 2:** Detection (*p*) and occupancy (ψ) explanatory covariates for the candidate models aimed at examining (i) the role of inter‐specific co‐occurrences among roe deer, red deer and wolf (M1, M2, M3), (ii) the impact of human presence (M4, M5, M6) and (iii) the effects of the hunting season (M7, M8, M9) on the species presence.

Model		Species	*p* covariates	ψ covariates
(i) Species co‐occurrences	M1	Roe	Visual + TRI + Trail	Forest + TRI + Roads
		Red	Visual + TRI + Trail	TRI + Buildings
		Wolf	Visual + TRI + Trail	TRI + Hum_passage
	M2	Roe	Visual + TRI + Trail	Forest + TRI + Roads
		Red	Visual + TRI + Trail	TRI + Buildings
		Wolf	Visual + TRI + Trail	TRI + Hum_passage
		Roe/Red		1
		Roe/Wolf		1
		Red/Wolf		1
	M3	Roe	Visual + TRI + Trail	Forest + TRI + Buildings
		Red	Visual + TRI + Trail	TRI + Buildings
		Wolf	Visual + TRI + Trail	TRI + Hum_passage
		Roe/Red		Open + Roads
		Roe/Wolf		Forest + Buildings
		Red/Wolf		Buildings
(ii) The impact of human presence	M4	Roe	Visual + TRI + Trail + Human	Forest + TRI + Roads
		Red	Visual + TRI + Trail + Human	TRI + Buildings
		Wolf	Visual + TRI + Trail + Human	TRI + Hum_passage
	M5	Roe	Visual + TRI + Trail + Human	Forest + TRI + Roads
		Red	Visual + TRI + Trail + Human	TRI + Buildings
		Wolf	Visual + TRI + Trail + Human	TRI + Hum_passage
		Roe/Red		1
		Roe/Wolf		1
		Red/Wolf		1
	M6	Roe	Visual + TRI + Trail + Human	Forest + TRI + Roads
		Red	Visual + TRI + Trail + Human	TRI + Buildings
		Wolf	Visual + TRI + Trail + Human	TRI + Hum_passage
		Roe/Red		Open + Roads
		Roe/Wolf		Forest + Buildings
		Red/Wolf		Buildings
(iii) The effects of hunting	M7	Roe	Visual + TRI + Trail + Hunting	Forest + TRI + Roads
		Red	Visual + TRI + Trail + Hunting	TRI + Buildings
		Wolf	Visual + TRI + Trail + Hunting	TRI + Hum_passage
	M8	Roe	Visual + TRI + Trail + Hunting	Forest + TRI + Roads
		Red	Visual + TRI + Trail + Hunting	TRI + Buildings
		Wolf	Visual + TRI + Trail + Hunting	TRI + Hum_passage
		Roe & Red		1
		Roe & Wolf		1
		Red & Wolf		1
	M9	Roe	Visual + TRI + Trail + hunting	Forest + TRI + Roads
		Red	Visual + TRI + Trail + hunting	TRI + Buildings
		Wolf	Visual + TRI + Trail + hunting	TRI + Hum_passage
		Roe & Red		Open + Roads
		Roe & Wolf		Forest + Buildings
		Red & Wolf		Buildings

*Note:* Roe = roe deer, Red = red deer, Wolf = wolf, Visual = visual clarity, trail = type of trail, forest/open = % of forest/open habitats in a 200 m buffer from the camera, TRI = terrain ruggedness index, Roads/Buildings = distance of the camera from the closest road/building, Hum_passage = human capture rate for each camera, Human = record of presence or absence of humans each day, Hunting = record of the days when hunting was active.

(i) Species co‐occurrence. The first set of 3 models aimed to evaluate the extent to which species co‐occurrence plays a role in explaining the occupancy of model species across the landscape (Table 2, i). We performed model selection for the detection and occupancy models of each species (detailed results are reported in Appendix A). M1, M2 and M3 assumed species‐specific *p* as a function of the visual clarity of the camera, TRI and whether the CT was on or off a trail, as performance indices. Conversely, ψ varied according to the species of interest, remaining constant in all three sets of models. Drawing from previous research and following model selection, M1 suggested that all three species occur independently, with roe deer marginal ψ modeled as a function of forest and TRI, due to the species' preference for glade‐like environments within forested areas (Lovari et al. 2017), and well‐documented avoidance of steep or rugged terrains, with TRI being one of the primary factors to lead habitat use in roe deer in similar areas (Wevers et al. 2021). Moreover, distance from roads was also selected as a human impact covariate, after model selection, and confirmed by previous studies where roe deer tended to avoid roadways in various habitats (Coulon et al. 2008; Bonnot et al. 2013). Red deer marginal ψ was modelled with TRI and distance from buildings. Red deer were expected to be mostly influenced by the distance from buildings since those tend to create openings in the forest canopy—the habitat within our study area is predominantly wooded due to the altitudinal range it covers—that trigger a transition to younger and smaller plants (McDowell et al. 2020), which can provide browsing and foraging opportunities (Porter et al. 2004). Wolf marginal ψ was modelled with TRI and the average number of hikers, as wolves usually tend to minimise their probability of encountering people, despite their use of analogous landscape features for movement, such as hiking trails and roads (Dickie et al. 2017). M2 replicated the marginal ψ as defined in M1 and further incorporated the hypothesis of constant pairwise dependence among species (i.e., without any covariate). In M3, the pairwise dependence among species was not kept constant but modelled with covariates, aiming to evaluate their single or combined impact. Following model selection based on ecological hypotheses, the probability that roe deer and red deer occur together was hypothesised to be affected by open areas and distance from roads. Both ungulates are known to seek for openings in the forest canopy for grazing, since those openings generally enhance foraging conditions by substantially increasing ground‐level plant biomass availability (Kuijper et al. 2009). At the same time, both species tend to avoid sources of human disturbance, particularly roads (Petridou et al. 2023; Putzu et al. 2014). Consequently, a higher co‐occurrence probability was expected in habitats with a greater percentage of open areas and farther from roads. The probability that roe deer and wolves occur together was expected to be affected by forests and distance from buildings. Both species use forests as refuge areas and avoid areas with stable human disturbance (Bonnot et al. 2013). Finally, the modelling of red deer and wolves' co‐occurrence was based on distance from buildings, reflecting the red deer's tendency to occupy areas closer to buildings in our study area (as indicated by the model selection process; Appendix A). It was expected that wolves would co‐occur in these areas if red deer were predominantly present.

(ii) The impact of human presence. In models M4, M5 and M6, *p* depended on the varying presence or absence of humans (i.e., across surveys), a factor revealed to significantly affect both prey and predators (Oberosler et al. 2017) (Table 2, ii). The underlying hypotheses regarding the occupancy process mirrored those of M1, M2 and M3, respectively, to confirm the first hypothesis regarding the relevance of species co‐occurrence patterns.

(iii) The effects of hunting. M7, M8 and M9 aimed to explore the potential effect of hunting on the target species. The ψ covariates align with the previously described hypotheses, while we introduced hunting days as a covariate in modeling *p*, expecting to find a negative relation with roe deer, which is the only game species in the study area (Table 2, iii). Once again, this set includes three models as already specified.

The candidate models were ranked using the Akaike Information Criterion (AIC; Burnham and Anderson 2002).

## 3 Results

Of the 60 CTs, two were not positioned due to high snow coverage, one was stolen, and one did not work properly. Consequently, the final dataset consisted of 56 CT sites and 6244 trap days. Of all the positioned cameras, 62% were positioned at the first random location, 28% at the second and 10% at the third. Moreover, 80% of the cameras were positioned during the initial 2 months of the sampling period (November: *n* = 22, December: *n* = 25, January: *n* = 8, February: *n* = 1, March: *n* = 2) and once positioned, they collected photo data until the end of the study. The mean of trap days was 112 ± 38 days. 20% of the total CTs were located inside the natural protected area and the remnant was in regulated hunting areas.

### 3.1 Naive Occupancy

The dataset included a total number of 35,571 pictures, cleaned from false triggers (i.e., blank photos). The detected animals are reported in Table 3. In particular, we obtained 2594 detections of roe deer across 52 sites, 1770 detections of humans at 29 sites, 332 detections of red deer at 21 sites and 124 detections of wolves at 23 sites.

**TABLE 3:** Total number of events per species captured with camera traps in Alpine valleys of the Western Italian Alps, from November 2021 to April 2022. Capture rate (i.e., number of events/sampling effort × 100) and naive occupancy (i.e., number of sites occupied/total sites) are also reported.

Species	Events	Capture rate	Naive ψ
Roe deer ( *Capreolus capreolus* )	2594	41.54	0.93
Human ( *Homo sapiens* )	1770	28.35	0.52
Wild boar ( *Sus scrofa* )	1485	23.78	0.80
Fox ( *Vulpes vulpes* )	693	11.10	0.71
Badger ( *Meles meles* )	466	7.46	0.57
Red deer ( *Cervus elaphus* )	332	5.32	0.37
Dog ( *Canis lupus familiaris* )	200	3.20	0.37
Martes sp.	138	2.21	0.55
Wolf ( *Canis lupus* )	124	1.99	0.41
Chamois ( *Rupicapra rupicapra* )	112	1.79	0.16
Other	532		
Total	8308		

*Note:* The category “Other” includes: Birds (events: *n* = 110), domestic cat (*n* = 60), red squirrel (*n* = 23), brown hare (*n* = 13), livestock (*n* = 1) and unidentifiable animals (i.e., out of focus or low‐quality picture, *n* = 187). Highlighted in grey are the species of interest for this research.

### 3.2 Model Selection

Overall, the models provided support for the significant relevance of including interspecific co‐occurrences of the three observed species. Based on AIC values, the best model of each distinct set resulted in being the one that incorporated species‐specific dependence in relation to covariates. In particular, (i) M3 (ΔAIC = 9.14) included environmental variables to model the species' ψ without accounting for human disturbance in explaining *p*, (ii) M6 obtained the same result (ΔAIC = 9.57) by considering human presence as an explanatory covariate for *p*, while (iii) M9 best fitted the data and performed best (ΔAIC = 0) by considering hunting days as an explanatory covariate for species *p*. The models compared with the AIC are displayed in Table 4 and the best model's estimated values of species‐specific occupancy and detection probabilities are reported in Table 5.

**TABLE 4:** AIC model comparison results. ΔAIC is the difference of each model's AIC.

Model	AIC	ΔAIC	Species	*p* covariates	ψ covariates
M9	7140.59	0	Roe Red Wolf Roe/Red Roe/Wolf Red/Wolf	Visual + TRI + Trail + Hunting Visual + TRI + Trail + Hunting Visual + TRI + Trail + Hunting	Forest + TRI + Roads TRI + Buildings TRI + Hum_passage Open + Roads Forest + Buildings Buildings
M7	7141.68	1.09	Roe Red Wolf	Visual + TRI + Trail + Hunting Visual + TRI + Trail + Hunting Visual + TRI + Trail + Hunting	Forest + TRI + Roads TRI + Buildings TRI + Hum_passage
M8	7145.22	4.63	Roe Red Wolf Roe/Red Roe/Wolf Red/Wolf	Visual + TRI + Trail + Hunting Visual + TRI + Trail + Hunting Visual + TRI + Trail + Hunting	Forest + TRI + Roads TRI + Buildings TRI + Hum_passage 1 1 1
M3	7149.73	9.14	Roe Red Wolf Roe/Red Roe/Wolf Red/Wolf	Visual + TRI + Trail Visual + TRI + Trail Visual + TRI + Trail	Forest + TRI + Roads TRI + Buildings TRI + Hum_passage Open + Roads Forest + Buildings Buildings
M6	7150.16	9.57	Roe Red Wolf Roe/Red Roe/Wolf Red/Wolf	Visual + TRI + Trail + Human Visual + TRI + Trail + Human Visual + TRI + Trail + Human	Forest + TRI + Roads TRI + Buildings TRI + Hum_passage Open + Roads Forest + Buildings Buildings
M1	7150.78	10.19	Roe Red Wolf	Visual + TRI + Trail Visual + TRI + Trail Visual + TRI + Trail	Forest + TRI + Roads TRI + Buildings TRI + Hum_passage
M4	7151.14	10.55	Roe Red Wolf	Visual + TRI + Trail + human Visual + TRI + Trail + human Visual + TRI + Trail + human	Forest + TRI + Roads TRI + Buildings TRI + Hum_passage
M2	7154.37	13.78	Roe Red Wolf Roe/Red Roe/Wolf Red/Wolf	Visual + TRI + Trail Visual + TRI + Trail Visual + TRI + Trail	Forest + TRI + Roads TRI + Buildings TRI + Hum_passage 1 1 1
M5	7154.75	14.16	Roe Red Wolf Roe/Red Roe/Wolf Red/Wolf	Visual + TRI + Trail + Human Visual + TRI + Trail + Human Visual + TRI + Trail + Human	Forest + TRI + Roads TRI + Buildings TRI + Hum_passage 1 1 1

*Note:* In grey the three best models of each set, which include interspecific dependence in relation to covariates (M3, M6, M9). M2, M5 and M8 include species co‐occurrence but assume constant pairwise dependence. M1, M4 and M7 assume that the three species occur independently. Roe = roe deer, Red = red deer, Wolf = wolf, Visual = visual clarity, Trail = type of trail, forest/open = % of forest/open habitats in a 200 m buffer from the camera, TRI = terrain ruggedness index, Roads/Buildings = distance of the camera from the closest road/building, Hum_passage = human capture rate for each camera, Human = record of presence or absence of humans each day, Hunting = record of the days when hunting was active.

**TABLE 5:** Occupancy (ψ) and detection (*p*) estimated values of the best model (M9), which accounts for hunting as an explanatory *p* covariate. The respective standard errors (SE) are also reported.

Species	Naive ψ	ψ	SE (ψ)	*p*	SE (*p*)
Roe deer	0.928	0.911	0.030	0.199	0.0012
Red deer	0.375	0.450	0.042	0.04	0.0003
Wolf	0.410	0.475	0.035	0.021	0.0002

**FIGURE 2:**
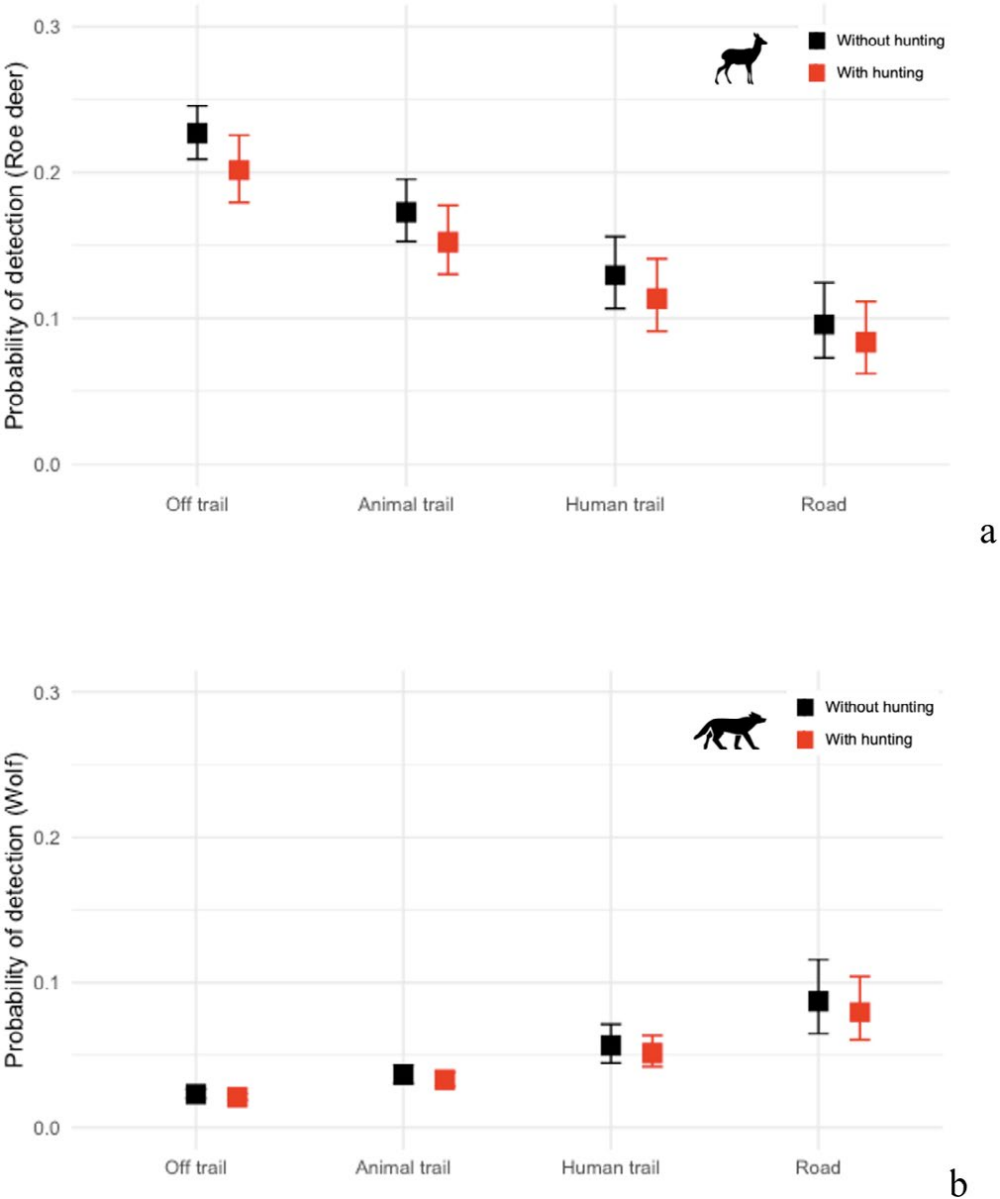
Detection probabilities (*p*) of roe deer (a) and wolf (b) in Alpine valleys of the Western Italian Alps as a function of camera's positioning on diverse categories of trails, when hunting is prohibited (black) and when it is active and regulated (red). Differential detectability is shown based on the camera trap (CT) location. CTs placed on trails were more likely to detect wolves, whereas those placed off‐trails were more likely to detect ungulates. Only roe deer's detection probability changed when hunting was added as a covariate (*p* < 0.001).

The detailed results of all the models, with indications of the significant covariates (i.e., *p* < 0.05), are reported in the Appendix (Tables A7, A8, A9). The top ranked model (M9; Table 6) showed that the detection of each species depended on the CT's positioning on a trail (Figure 2). Roe deer (*β* = −0.339, *p* < 0.001) and red deer (*β* = −1.078, *p* < 0.001) were less likely to be detected on trails, whereas wolves (*β* = 0.470, *p* < 0.001) were more likely to be detected on trails. The visual clarity index significantly influenced roe deer detection (*β* = 0.410, *p* < 0.001) but had no effect on the other species. Moreover, roe deer detection depended on hunting days, showing that the species was less likely to be detected when these were considered (*β* = −0.151, *p* < 0.001), while red deer and wolves detection was not affected by hunting days. The marginal occupancy of the two ungulate species depended on TRI. Both roe deer (*β* = −5.301, *p* = 0.018) and red deer (*β* = −1.970, *p* = 0.013) were less likely to occupy areas with increasing values of TRI. On the contrary, wolves were more likely to occupy areas where TRI reported higher values (*β* = 1.480, *p* = 0.016). In terms of co‐occurrences, roe deer and red deer were more likely to occur together when the percentage of open areas decreased (*β* = −1.289, *p* = 0.030). The other selected variables did not report any significant effect on the species co‐occurrences, despite contributing to the improvement of the model's performance.

**TABLE 6:** MSOM results for each explanatory covariate of the best model M9, evaluating species pairwise dependence modelled with covariates, with detection probability modelled as a function of the hunting period.

Species (marginal)	Covariate	Estimate (*β*)	SE	*p*	Species (co‐occurrence)	Covariate	Estimate (*β*)	SE	*p*
Roe deer	Intercept *p* Visual Trail TRI Hunting	−1.568 0.410 −0.339 −0.610 −0.151	0.040 0.044 0.038 0.042 0.040	< 0.001* < 0.001* < 0.001* < 0.001* < 0.001*	Roe‐Red	Intercept ψ Open Roads	−1.747 −1.289 1.059	1.861 0.592 0.634	0.348 0.030* 0.095
	Intercept ψ Forest TRI Roads	6.294 0.837 −5.301 2.000	2.748 0.958 2.231 1.215	0.022 0.382 0.018* 0.100	Roe‐Wolf	Intercept ψ Forest Buildings	1.640 −0.474 0.893	1.497 0.529 0.543	0.273 0.370 0.100
Red deer	Intercept *p* Visual Trail TRI Hunting	−3.445 0.100 −1.078 0.179 −0.034	0.148 0.115 0.169 0.118 0.137	< 0.001* 0.385 < 0.001* 0.127 0.803	Red‐Wolf	Intercept ψ Buildings	2.243 −0.182	1.309 1.498	0.087 0.903
	Intercept ψ TRI Buildings	−0.407 −1.970 −2.682	1.799 0.796 1.428	0.821 0.013* 0.060					
Wolf	Intercept *p* Visual Trail TRI Hunting	−4.050 0.270 0.469 0.128 0.096	0.190 0.237 0.119 0.148 0.154	< 0.001* 0.255 < 0.001* 0.385 0.532					
	Intercept ψ TRI Hum_passage	−1.438 1.480 5.735	1.502 0.612 2.853	0.338 0.016* 0.044*					

*Note:* Statistically significant results are highlighted with an asterisk (*) at *p* < 0.05. Roe = roe deer, Red = red deer, Visual = visual clarity, Trail = type of trail, forest/open = % of forest/open habitats in a 200 m buffer from the camera, TRI = terrain ruggedness index, Roads/Buildings = distance of the camera from the closest road/building, Hum_passage = human capture rate for each camera, Hunting = record of the days when hunting was active.

## 4 Discussion

Our study revealed that modelling species co‐occurrence patterns plays a crucial role in influencing the occupancy probabilities of ungulates and wolves in Italian Alpine valleys where they coexist alongside human presence and activities. Pairwise species co‐occurrences were found to exert a stronger influence on occupancy dynamics than environmental factors alone, highlighting the ecological interdependence within species in this landscape. Roe deer emerged as the most widespread species, with the highest frequency of recorded events, while wolves and red deer demonstrated notable but lower occupancy rates. The study design based on random placement of CT was important to guarantee comparison among species detections, as on‐trail CTs were more likely to detect wolves but less likely to detect ungulates. Notably, the hunting season had a pronounced effect on roe deer, significantly reducing their detectability, while red deer and wolves remained unaffected, emphasizing the differential sensitivity of species to anthropogenic disturbances.

Previous research has demonstrated that the placement of CTs along roads/trails as opposed to off roads/trails results in markedly different representations of mammal communities (Di Bitetti et al. 2014). Additionally, assessments of relative abundance have shown a bias toward large carnivores when using on‐trail CT placement methods, whereas unbiased estimates are obtained through random placement designs (Tanwar et al. 2021). Coherently, our results revealed evidence of differential detectability based on CT location. CTs placed on trails were more likely to detect wolves, whereas those placed off‐trails were more likely to detect ungulates. This finding aligns with current knowledge of wolves' movement strategies and their tendency to use linear features as convenient travel routes across their territory (Dickie et al. 2017). The estimated occupancy of wolves in our study area was 0.475, which is an arguably significant proportion considering the species' elusive nature and extensive home ranges (Marucco et al. 2023). Red deer exhibited similar values (ψ = 0.450) and roe deer the highest (ψ = 0.911). Therefore, our use of random CT placement confirmed the effectiveness of this sampling strategy in detecting multiple species, thereby avoiding potential biases toward carnivores or ungulates that might have arisen if CTs were solely placed on or off trails, respectively. In our study, we supplemented the random placement of cameras with MSOMs to address imperfect detection and account for the limitations that can affect the species detectability (such as the CT's field of view, the triggering mechanism, the behaviour of the species or the environmental conditions). Failure to account for this issue could result in biased estimates of occupancy, leading to incorrect conclusions about the distribution and ecology of species (Bailey et al. 2014).

In terms of marginal occupancy, both ungulates were more likely to occupy areas with lower terrain ruggedness values. This suggests a movement strategy aimed at maximizing energy efficiency by occupying gentler slopes and avoiding rugged terrain, as also demonstrated in previous studies (Wevers et al. 2021; Lone et al. 2014). Our findings showed a positive relationship between wolf marginal occupancy and human passage that needs to be carefully interpreted. Wolves are known to use linear features, such as trails and roads, as efficient travel routes within their territories (Dickie et al. 2017). However, these trails also coincide with human movement patterns, particularly in the mountainous area where our study was conducted, which has an extensive network of hiking trails. Hence, the positive effect for the same type of passage has to be attributed to a shared preference for trail‐based movement and not to a surrounding high‐quality habitat for wolves (Whittington et al. 2005). The current study did not explore the hypothesis of temporal segregation, as temporal patterns were not investigated. Previous studies have examined the temporal patterns of wolves, revealing that the species is predominantly active at night, while humans tend to be active during the day, enabling wolves to utilize areas that are heavily used by humans, trails included, at different times (Petridou et al. 2023; Kusak et al. 2005).

Considering the importance of taking a multi‐species approach, particularly in habitats where multiple species engage in prey–predator or competitive relationships (Amir et al. 2022), our modelling analysis demonstrated that accounting for co‐occurrence patterns improved model performance. Despite the valuable insights provided by single species relationships with their environment, our study demonstrates that employing MSOMs allows for a more accurate representation of carnivore and ungulate occurrences in a shared habitat. Indeed, in each of the three sets of our analysis, the model incorporating species co‐occurrence consistently outperformed the others. We selected explanatory variables for modelling species co‐occurrence based on previous research indicating their potential effect. This choice allowed us to improve models' performances, despite yielding few statistically significant *p*‐values. Indeed, in an information‐theoretic framework (i.e., AIC) the *p*‐values associated with covariates are less critical than the overall model rankings, enabling the species co‐occurrence models to provide valuable information when modelling the occupancy of predator and prey species.

Contrary to our expectation that ungulates would co‐occur in open areas due to forage availability, our findings indicate that roe deer and red deer are more likely to occur together when open areas decrease. This observation can, in fact, be explained by the characteristics of our study area, where broad‐leaved forests and relatively low elevations provide a supply of grasses that ungulates can exploit without leaving the forest (Heurich et al. 2015). Therefore, this finding supports the role of forests as refuges for ungulates when forage is available, as they offer both access to cover and a safer option to avoid human activity, as also noted in previous studies (Heurich et al. 2015; Padié et al. 2015).

Nevertheless, interspecific co‐occurrences are not limited to wild species sharing a habitat, as humans actively participate in these dynamics, particularly within the highly anthropized landscapes of Europe, which include regions like the Alps, with tourist‐heavy human populations. Predator–prey interactions represent a continuous competition for space wherein prey minimize and predators maximize spatial overlap (Muhly et al. 2011). Within this ecological balance, human activities directly influence animal distribution by engaging in their own competition for space (Corradini et al. 2021). Worldwide, spatiotemporal responses aimed at avoiding direct contact with humans are commonly observed (Bonnot et al. 2013).

Our investigation revealed that the hunting season significantly reduced the likelihood of detecting roe deer but did not affect either red deer or wolves. Since roe deer are the only game species among the three in our study area, they are potentially more sensitive to hunting periods. Cote et al. (2017) showed that roe deer reduce their movement during the hunting season, since moving across patches in fragmented habitats is highly risky. Similarly, Bonnot et al. (2013) observed a behavioral shift in roe deer in response to hunting disturbance. They reduced their daytime occupancy of high‐crop areas (i.e., forage‐rich habitats), increasing utilization of woodlands, as a strategic response to minimize vulnerability.

In human‐dominated landscapes, predator conservation becomes a complex challenge in which hunting is fully involved, as demonstrated by our results. Indeed, human‐wildlife conflicts arise from a negative perception of wolves by hunters, which reflects a competitive situation (Bisi et al. 2010). Hunters tend to attribute a perceived decline in roe deer populations to the recolonization of the Alps by wolves. However, our findings suggest that this perceived decrease may be attributed to the ungulate's behavioral response to predatory pressure, rather than an actual decline. Moreover, prey and predator have coexisted in this area for almost three decades (Marucco et al. 2023) and roe deer were the most widespread and most frequently detected species in our study.

The diversity of prey–predator and human‐wildlife co‐occurrence patterns in a shared habitat underscores the necessity for a comprehensive range of conservation policies to effectively address conflicting objectives and trade‐offs (Linnell et al. 2020). Our findings suggest that the use of habitat by wildlife should not only be examined in relation to specific environmental variables since species coexist with each other and with human activities, and their co‐occurrences cannot be overlooked. Roe deer, a species sensitive to hunting risk (Norum et al. 2015), was affected in their detectability by the hunting season in our study, highlighting the importance of this anthropogenic disturbance in understanding the species' response to its environment. Our insights highlight the importance of considering anthropogenic influences when analyzing predator–prey shared habitats, in order to define management solutions to optimize human‐wildlife coexistence, especially in the highly anthropogenic landscapes of Europe.

## Author Contributions

**F. Rolle:** data curation (lead), formal analysis (equal), methodology (equal), software (lead), writing – original draft (lead), writing – review and editing (equal). **M. V. Boiani:** formal analysis (equal), methodology (equal), software (equal), supervision (supporting), writing – review and editing (equal). **L. Fardone:** methodology (equal), writing – review and editing (equal). **F. Gaydou:** methodology (equal), writing – review and editing (equal). **M. Macario:** methodology (equal), writing – review and editing (equal). **F. Parentela:** methodology (equal), writing – review and editing (equal). **V. Ruco:** methodology (equal), supervision (supporting), writing – review and editing (equal). **D. Sigaudo:** conceptualization (equal), methodology (lead), supervision (lead), writing – review and editing (equal). **F. Marucco:** conceptualization (lead), funding acquisition (lead), methodology (equal), project administration (lead), resources (equal), supervision (lead), writing – review and editing (lead).

## Conflicts of Interest

The authors declare no conflicts of interest.

## Figures and Tables

**FIGURE A1:**
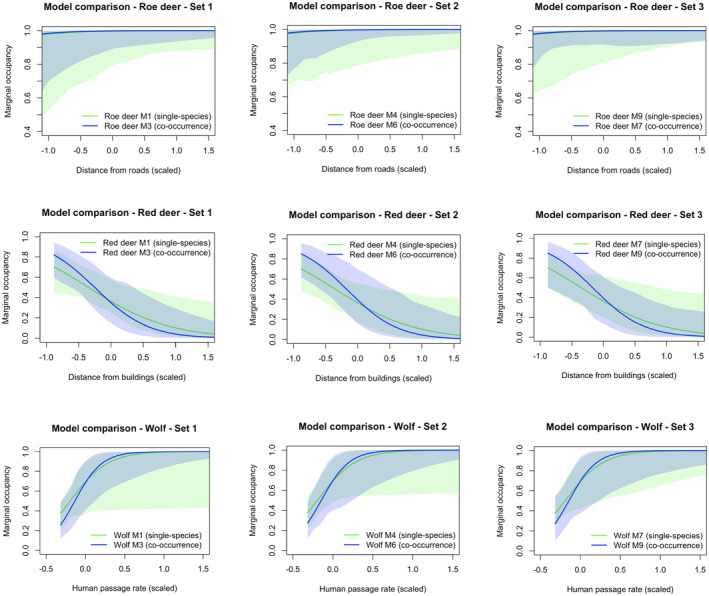
Effect size of covariates from the single‐species and multi‐species occupancy models. The green line represents the predicted relationship from the single‐species models (M1, M4 and M7), and the blue line represents the predicted relationship from the multi‐species model (M3, M6 and M9). The three sets are as follows: Set 1—Species co‐occurrence (Models M1‐3); Set 2—The impact of human presence (Models M4‐6); Set 3—The effects of hunting (Models M7‐9). The first row corresponds to roe deer, the second row to red deer, and the third row to wolf.

## Data Availability

Data associated with this manuscript is archived on Mendeley Data at https://data.mendeley.com/datasets/362y2t8fzd/1

## Appendix A

### The Model Selection Process for Multi‐Species Occupancy Modelling

We evaluated a series of candidate models to select the most informative covariates influencing the detection and occupancy probabilities of each species of interest (roe deer, red deer and wolf) to build the multi‐species occupancy models. Model selection was based on the Akaike Information Criterion (AIC; Burnham and Anderson 2002). The analyses were conducted in the software R 4.2.2 (R Core Team 2022), using the packages *unmarked* (Fiske and Chandler 2011) and *MuMIn* (Bartoń 2024). The results of the model selection process for each species are reported in Tables A1 (Detection) and A2, A3, A4 (Occupancy).

#### Details of the Model Selection Process for the Detection Models

For the detection, the tested models included combinations of the variables *Trail*, *TRI* (Terrain Ruggedness Index), and *Visual clarity*. The definition of the covariates and how those were calculated are reported in the main manuscript in the *Covariate selection* section of the *Methods* and summarised in Table 1 of the main manuscript.

##### Roe Deer

The top model for the detection section of roe deer was the full model *Trail + TRI + Visual*, which reported the lowest AIC value and a ΔAIC exceeding 80 with the second‐ranked model, indicating it was unequivocally the most informative model among those tested (Table A1). Therefore, the inclusion of all three detection covariates in the top model indicated that these factors are collectively important for roe deer detection. Among these, *TRI* appears particularly informative, as its inclusion in the model consistently improved performance compared to models that excluded it (Table A1).

##### Red Deer

The top four models had ΔAIC values under 2, indicating they all received reasonable support: (1) *Trail* (AIC = 984.54, AIC weight = 0.34), (2) *Trail + TRI* (AIC = 984.56, ΔAIC = 0.01, AIC weight = 0.34), (3) *Trail + TRI + Visual* (AIC = 985.91, ΔAIC = 1.37, AIC weight = 0.17), (4) *Visual + Trail* (AIC = 986.38, ΔAIC = 1.83, AIC weight = 0.14) (Table A1). Together, these models account for the totality of the cumulative AIC weight, highlighting *Trail* as the most important covariate, with *TRI* and *Visual clarity* providing additional but more limited contributions in certain combinations. Since ignoring these contributions could result in underestimating the uncertainty and variability in the detection process, we performed model averaging across the top four models with ΔAIC < 2. The model‐averaged coefficients were computed from these models, and the averaged results highlighted *Trail* as having a strong and significant negative effect on detection probability (*β* = −1.04, *p* < 0.001). While their contributions are smaller, *TRI* and *Visual clarity* reflect potential interactions with *Trail* that are not captured in isolation. Therefore, we used the full model for the successive analyses as it provided a more comprehensive view by incorporating uncertainty associated with the secondary variables (Cum.Wt = 0.86).

##### Wolf

The two best‐supported models, *Trail + TRI* (AIC 632.84) and *Trail + TRI + Visual* (AIC 632.93) (Table A1), had ΔAIC values of < 2, indicating substantial support for both. These two models together accounted for nearly all of the model weight, suggesting that they were the most informative for explaining detection probability in wolves. To account for model selection uncertainty, we performed model averaging on these top two models with ΔAIC < 2. The model‐averaged coefficients revealed that *Trail* had a significantly positive effect on detection probability (*β* = 0.57, *p* < 0.001). However, *Trail* is not the most informative covariate on its own. Indeed, the model *Trail* alone showed a poor fit (ΔAIC = 33.76), indicating that *Trail* by itself is not sufficient to explain wolf detection. It is, however, an important predictor when combined with *TRI* and *Visual clarity* in the full model.

**TABLE A1:** Model selection results for roe deer, wolf, and red deer individual detection models. Detection covariates for each model are listed in the first column, with occupancy held constant at 1.

Roe deer	*K*	AIC	ΔAIC	AICWt	Cum.Wt	*β*
Trail + TRI + Visual	5	5563.86	0	1	1	*β*_Trail_ = −0.34* *β*_TRI_ = −0.59* *β*_Visual_ = 0.41*
TRI + Visual	4	5646.79	82.92	0	1	*β*_Visual_ = 0.33* *β*_TRI_ = −0.66*
Trail + TRI	4	5747.83	183.97	0	1	*β*_Trail_ = −0.20* *β*_TRI_ = −0.72*
TRI	3	5775.76	211.90	0	1	*β*_TRI_ = −0.74*
Trail + Visual	4	5784.72	220.86	0	1	*β*_Visual_ = 0.36* *β*_Trail_ = −0.41*
Trail	3	5858.81	294.95	0	1	*β*_Trail_ = −0.35*
Visual	3	5916.18	352.32	0	1	*β*_Visual_ = 0.24*
Null	2	5951.43	387.57	0	1	

Red Deer	*K*	AIC	ΔAIC	AICWt	Cum.Wt	*β*
Trail	3	984.54	0	0.34	0.34	Averaged *β*_Trail_ = −1.04* *β*_TRI_ = 0.08 *β*_Visual_ = 0.02
Trail + TRI	4	984.56	0.01	0.34	0.69	
Trail + TRI + Visual	5	985.91	1.37	0.17	0.86	
Trail + Visual	4	986.38	1.83	0.14	1	
Visual	3	1040.59	56.05	0	1	*β*_Visual_ = −0.10
TRI	3	1040.75	56.20	0	1	*β*_TRI_ = 0.10
Visual + TRI	4	1042.24	57.69	0	1	*β*_Visual_ = −0.08 *β*_TRI_ = 0.08
Null	2	1183.95	199.40	0	1	

WOLF	*K*	AIC	ΔAIC	AICWt	Cum.Wt	*β*
Trail + TRI	4	632.84	0	0.51	0.51	Averaged *β*_Trail_ = 0.57* *β*_TRI_ = 0.20 *β*_Visual_ = 0.14
Trail + TRI + Visual	5	632.93	0.10	0.49	1	
Visual	3	647.37	14.54	0.00	1	*β*_Visual_ = 0.68*
TRI + Visual	4	649.15	16.32	0.00	1	*β*_Visual_ = 0.69* *β*_TRI_ = 0.06
Trail + Visual	4	662.96	30.12	0.00	1	*β*_Visual_ = 0.463* *β*_Trail_ = 0.478*
Trail	3	666.60	33.76	0.00	1	*β*_Trail_ = 0.591*
Trail*TRI	5	667.42	34.59	0.00	1	*β*_Trail_ = 0.601* *β*_TRI_ = 0.204
TRI	3	690.81	57.97	0.00	1	*β*_TRI_ = 0.185
Null	2	690.94	58.10	0.00	1	

*Note:* The Akaike Information Criterion (AIC) is used for model comparison, ΔAIC represents the difference in AIC values compared to the top model, AICWt indicates the relative likelihood of each model, and Cum.Wt is the cumulative weight of the models. Statistically significant results are highlighted with an asterisk (*) at *p* < 0.05. Trail = CT positioned on/off a trail, TRI = Terrain Ruggedness Index, Visual = visual clarity.

#### Details of the Model Selection Process for the Occupancy Models

For each species, we tested individual occupancy models using covariate combinations informed by previous research that identified potential effects on the species of interest. Human disturbance covariates included (i) distance from roads, (ii) distance from buildings, and (iii) the average number of humans recorded by each CT per day. Environmental covariates included (i) forest cover and (ii) the Terrain Ruggedness Index (TRI). All three human disturbance covariates were included in each individual occupancy model, and model selection was performed to identify the most informative covariates for each species.

##### Roe Deer

The habitat within our study area is predominantly wooded due to the altitudinal range it covers, with limited expansion of areas with scarce vegetation or pastures. Roe deer has been identified as a wood‐dependent species (Morellet et al. 2011; Hewison et al. 2001; Lovari and San Josè 1997) and its small body size (averaging 27.7 kg for males and 26.7 kg for females; Loison et al. 1999) is suitable for moving through densely vegetated habitat conditions (Hansson 1994). Therefore, the percentage of forest cover was included as a covariate for modelling roe deer marginal occupancy, as it was expected to have a significant effect on the species. Moreover, roe deer has been positively associated with gentle slopes and avoidance of rugged terrain (Wevers et al. 2021; Lone et al. 2014), as a reflection of a movement strategy aimed at maximising energy efficiency (Petridou et al. 2023). Therefore, TRI was used as a covariate as one of the primary factors to lead habitat use in the species (Wevers et al. 2021; Lone et al. 2014). Additionally, roe deer are particularly sensitive to human presence and previous studies revealed they often avoid sources of disturbance such as roads, buildings, agricultural land and recreational activities (Petridou et al. 2023; Bonnot et al. 2013; Coulon et al. 2008). Therefore, distances from roads, buildings and human presence were all tested and used as covariates to represent indicators of major human disturbance to the species. Moreover, the effects of infrastructure on roe deer occupancy can be influenced by the immediate environment, as roe deer tolerate close proximity to settlements when they can seek refuge in forested habitats during the day (Bonnot et al. 2013). Hence, we tested both forest cover alongside anthropogenic variables in our model selection process.

Based on these previous ecological findings, we performed model selection for roe deer marginal occupancy as shown in Table A2. The results revealed that the top model's most informative variable was *TRI* (*β*_TRI_ = −4.28; confirming roe deer avoidance of steep terrains), along with *distance from roads* (*β*_Roads_ = 1.90) and *forest* cover (*β*_Forest_ = 0.12), providing the best explanation for roe deer occupancy. Conversely, models incorporating additional variables, such as *distance from buildings* or *human passage*, performed poorly (ΔAIC > 2), and single‐variable models showed no support (AIC weights ≈ 0). Consequently, the selected human disturbance covariate for roe deer was *distance from roads*, along with the environmental variables *TRI* and *Forest*.

**TABLE A2:** Model selection results for roe deer individual occupancy models. Occupancy covariates for each model are listed in the first column, with detection held constant at 1.

Roe deer	*K*	AIC	ΔAIC	AICWt	Cum.Wt	*β*
TRI + Roads	4	5934.19	0	0.54	0.54	Averaged *β*_TRI_ = −4.28* *β*_Roads_ = 1.90 *β*_Forest_ = 0.12
TRI + Forest + Roads	5	5936.17	1.98	0.20	0.75	
TRI + Forest + Roads Roads*Forest	6	5936.91	2.71	0.14	0.89	*β*_TRI_ = −4.44* *β*_Forest_ = −0.01 *β*_Roads_ = 1.91. *β*_Forest:Roads_ = 1.08
TRI	3	5938.28	4.09	0.07	0.96	*β*_TRI_ = −2.68*
TRI + Buildings	4	5939.94	5.74	0.03	0.99	*β*_TRI_ = −2.67* *β*_Buildings_ = −0.43
TRI + Forest+Buildings	5	5941.82	7.62	0.01	1	*β*_TRI_ = −2.54* *β*_Forest_ = 0.25 *β*_Buildings_ = −0.25
TRI + Forest+Hum_passage	5	5941.85	7.66	0.01	1	*β*_TRI_ = −2.45* *β*_Forest_ = 0.37 *β*_Hum_passage_ = 0.54
Hum_passage	3	5952.56	18.36	0	1	*β*_Hum_passage_ = 1.45
Forest	3	5952.64	18.45	0	1	*β*_Forest_ = 0.38
Roads	3	5952.95	18.76	0	1	*β*_Roads_ = 0.37

*Note:* The Akaike Information Criterion (AIC) is used for model comparison, ΔAIC represents the difference in AIC values compared to the top model, AICWt indicates the relative likelihood of each model, and Cum.Wt is the cumulative weight of the models. Statistically significant results are highlighted with an asterisk (*) at *p* < 0.05, and the dot (.) indicates a *p*‐value between 0.05 and 0.1. TRI = Terrain Ruggedness Index, Forest = forest cover, Roads = distance from roads, Buildings = distance from buildings, Hum_passage = average number of humans captured by each CT per day.

##### Red Deer

The individual occupancy models for red deer included all anthropogenic covariates used for roe deer, except for human passage. This covariate had a value of 0 in 43% of the cameras that detected red deer at least once. Notably, only 4 of these cameras were located on trails, which are primarily used by humans and rarely by red deer. Additionally, red deer events were relatively infrequent compared to other wildlife species. As a result, human passage was excluded as an explanatory variable in this case, as it does not interact meaningfully with red deer detections.

Based on the characteristics of our study area, we expect red deer occupancy to be primarily affected by the distance from buildings, also considering previous studies that revealed that human settlements can create edges or boundaries in the landscape, potentially offering more suitable habitat for red deer (Etter et al. 2002; Porter et al. 2004). Specifically, settlements can create openings in the forest canopy, facilitating a transition to younger and smaller plants (McDowell et al. 2020), which may provide enhanced browsing and foraging opportunities.

Our model testing process confirmed this hypothesis and it is shown in Table A3. The covariate *Buildings* was the most informative one and together with *TRI* and *Forest*, it makes the second best model. The two top models had ΔAIC < 2, so we performed model averaging, resulting in *Buildings* having a significant and negative effect on red deer occupancy (*β* = −1.63, *p* < 0.05). Therefore, the human disturbance covariate selected for red deer was buildings, along with the environmental variables *TRI* and *Forest*.

Our model testing process confirmed this hypothesis, as shown in Table A3. The covariate *Buildings* was the most informative, and together with *TRI* and *Forest*, it formed the second‐best model. Both top models had ΔAIC < 2, so we performed model averaging, which indicated that *Buildings* had a significant negative effect on red deer occupancy (*β* = −1.63, *p* < 0.05). Consequently, the selected human disturbance covariate for red deer was *Buildings*, along with the environmental variables *TRI* and *Forest*.

**TABLE A3:** Model selection results for red deer individual occupancy models. Occupancy covariates for each model are listed in the first column, with detection held constant at 1.

Red deer	*K*	AIC	ΔAIC	AICWt	Cum.Wt	*β*
Buildings	3	1028.59	0	0.57	0.57	Averaged *β*_TRI_ = −0.58 *β*_Forest_ = 0.60 *β*_Buildings_ = −1.63*
Buildings + TRI + Forest	5	1029.32	0.73	0.40	0.97	
TRI + Roads	4	1036.27	7.68	0.01	0.99	*β*_TRI_ = −0.61 *β*_Roads_ = 0.91*
TRI + Forest + Roads	5	1037.31	8.72	0.01	0.99	*β*_TRI_ = −0.70 *β*_Forest_ = 0.34 *β*_Roads_ = 0.84*
Forest + Roads	4	1038.71	10.12	0	1	*β*_Forest_ = 0.22 *β*_Roads_ = 0.53
Forest	3	1039.68	11.08	0	1	*β*_Forest_ = 0.39
Buildings + TRI	4	1136.39	107.80	0	1	*β*_Buildings_ = −45.61 *β*_TRI_ = −8.43
TRI + Forest	4	1165.67	137.08	0	1	*β*_TRI_ = −13.0 *β*_Forest_ = 31.10

*Note:* The Akaike Information Criterion (AIC) is used for model comparison, ΔAIC represents the difference in AIC values compared to the top model, AICWt indicates the relative likelihood of each model, and Cum.Wt is the cumulative weight of the models. Statistically significant results are highlighted with an asterisk (*) at *p* < 0.05 and the dot (.) indicates a *p*‐value between 0.05 and 0.1. TRI = Terrain Ruggedness Index, Forest = forest cover, Roads = distance from roads, Buildings = distance from buildings.

##### Wolf

Wolves are known to use linear features, such as hiking trails, as travel routes, offering convenient pathways across their territory (Dickie et al. 2017; Whittington et al. 2005). However, these trails also coincide with human movement patterns, particularly in the mountainous area where our study was conducted, which has an extensive network of hiking trails. Therefore, wolf occupancy was modelled as a function of distance from roads and human passage to account for this movement pattern, which is significant for wolves, as they are more likely to be detected on trails, whereas ungulates off trail (Table A4).

The best model for wolves included *distance from roads* and *human passage* as the most informative variables, both having positive effects. These variables account for the shared patterns of human movement (e.g., trails) while also reflecting the wolves' tendency to minimise encounters with people (Dickie et al. 2017), avoiding areas where roads are concentrated, such as valley floors. The top model had a ΔAIC value exceeding 10 compared to the second‐ranked model, indicating it was clearly the most informative. Therefore, the selected human disturbance covariates for wolves were *distance from roads* and *human passage*.

**TABLE A4:** Model selection results for wolf individual occupancy models. Occupancy covariates for each model are listed in the first column, with detection held constant at 1.

WOLF	*K*	AIC	ΔAIC	AICWt	Cum.Wt	*β*
Roads+Hum_passage	4	645.24	0	0.99	0.99	*β*_Roads_ = 0.65 *β*_Hum_passage_ = 3.87
TRI + Roads	4	655.44	10.20	0.01	1	*β*_TRI_ = 0.19 *β*_Roads_ = 0.45
TRI + Roads + Hum_passage	5	669.43	24.19	0	1	*β*_TRI_ = 54.6 *β*_Roads_ = −17.2 *β*_Hum_passage_ = 35.5
Forest	3	692.94	47.70	0	1	*β*_Forest_ = −1.72*
TRI + Buildings	4	694.94	49.70	0	1	*β*_TRI_ = 6.38 *β*_Buildings_ = −1.86

*Note:* The Akaike Information Criterion (AIC) is used for model comparison, ΔAIC represents the difference in AIC values compared to the top model, AICWt indicates the relative likelihood of each model, and Cum.Wt is the cumulative weight of the models. Statistically significant results are highlighted with an asterisk (*) at *p* < 0.05, and the dot (.) indicates a *p*‐value between 0.05 and 0.1. TRI = Terrain Ruggedness Index, Forest = forest cover, Roads = distance from roads, Buildings = distance from buildings, Hum_passage = average number of humans captured by each CT per day.

#### Details of the Model Selection Process for the Multi‐Species Occupancy Models

The initial step of model selection, described previously, involved identifying the best set of explanatory variables for each species independently. When transitioning to multi‐species occupancy models, it was necessary to account for co‐occurrence effects, as these can significantly contribute to explaining the observed patterns in the data. Consequently, we performed an additional round of model selection incorporating both the predictors identified in the single‐species models and terms representing co‐occurrence hypotheses.

In the multi‐species context, the best predictors for some species differed from those identified in single‐species models, reflecting the influence of co‐occurrence among different species and shared environmental responses on occupancy patterns. These results show the importance of integrating model selection when modeling multiple species, as this can reveal dynamics not apparent in single‐species analyses and provide a more comprehensive understanding of the factors driving occupancy.

The probability that roe deer and red deer occur together was modelled with open areas and distance from roads. Both ungulates are known to seek openings in the forest canopy for grazing, since those openings generally enhance foraging conditions for ungulates by promoting a substantial increase in ground‐level plant biomass availability (Kuijper et al. 2009) and tend to avoid sources of human disturbance, particularly roads (Petridou et al. 2023; Putzu et al. 2014). The probability that roe deer and wolves occur together was modelled with forests and distance from buildings, as both species tend to use forests as refuge/hiding areas (Bonnot et al. 2013). Finally, the modelling of red deer and wolves' co‐occurrence was based on distance from buildings, reflecting the first choice of red deer to select for areas closer to buildings, as described above.

Model *M_b* modeled wolf occupancy using the top models from the individual model selection process of the species. Models *M_d* and *M_c* modeled red deer occupancy using the first and second top models from the individual selection process (*Buildings* and *TRI + Buildings + Forest*, respectively). Model *M_e* modeled occupancy for each species with the relative top models identified in the previous individual occupancy selection: roe deer (*Forest + TRI + Roads*), red deer (*TRI + Buildings + Forest*) and wolf (*Roads + Human passage*). However, this model had a ΔAIC value exceeding 6 compared to the top‐ranked model, and the other models derived from the individual selection process also showed ΔAIC > 2. Therefore, model *M_a* was identified as the most informative and was used in all subsequent analyses (Table A5).

**TABLE A5:** Model selection for the multi‐species occupancy models based on AIC.

Model	AIC	ΔAIC	Species	*p* covariates	ψ covariates
M_a	7149.73	0	Roe Red Wolf Roe/Red Roe/Wolf Red/Wolf	Visual + TRI + Trail Visual + TRI + Trail Visual + TRI + Trail	Forest + TRI + Roads TRI + Buildings TRI + Hum_passage Open + Roads Forest + Buildings Buildings
M_c	7151.34	1.61	Roe Red Wolf Roe/Red Roe/Wolf Red/Wolf	Visual + TRI + Trail Visual + TRI + Trail Visual + TRI + Trail	Forest + TRI + Roads Forest + TRI + Buildings TRI + Hum_passage Open + Roads Forest + Buildings Buildings
M_b	7154.41	4.68	Roe Red Wolf Roe/Red Roe/Wolf Red/Wolf	Visual + TRI + Trail Visual + TRI + Trail Visual + TRI + Trail	Forest + TRI + Roads TRI + Buildings Roads + Hum_passage Open + Roads Forest + Buildings Buildings
M_e	7156.02	6.29	Roe Red Wolf Roe/Red Roe/Wolf Red/Wolf	Visual + TRI + Trail Visual + TRI + Trail Visual + TRI + Trail	Forest + TRI + Roads Forest + TRI + Buildings Roads + Hum_passage Open + Roads Forest + Buildings Buildings
M_d	7156.13	6.40	Roe Red Wolf Roe/Red Roe/Wolf Red/Wolf	Visual + TRI + Trail Visual + TRI + Trail Visual + TRI + Trail	Forest + TRI + Roads Buildings TRI + Hum_passage Open + Roads Forest + Buildings Buildings

*Note:* ΔAIC is the difference of each model's AIC. In grey the three best models, used for the analyses in the main manuscript, which includes species co‐occurrence dependence in relation to covariates. Roe = roe deer, Red = red deer, Wolf = wolf, Visual = visual clarity, Trail = CT positioned on/off a trail, forest/open = % of forest cover/open habitats, TRI = Terrain Ruggedness Index, Roads/Buildings = distance of the CT from the closest road/building, Hum_passage = human capture rate for each CT.

##### Other Tested Models

The additional models reported in Table A6 focus on a wider exploration of the co‐occurrence variables.

**TABLE A6:** Continuation of model selection for the multi‐species occupancy models based on AIC. ΔAIC is the difference of each model's AIC.

Model	AIC	ΔAIC	Species	*p* covariates	ψ covariates
M_a	7149.73	0	Roe Red Wolf Roe/Red Roe/Wolf Red/Wolf	Visual + TRI + Trail Visual + TRI + Trail Visual + TRI + Trail	Forest + TRI + Roads TRI + Buildings TRI + Hum_passage Open + Roads Forest + Buildings Buildings
M_f	7153.41	3.68	Roe Red Wolf Roe/Red Roe/Wolf Red/Wolf	Visual + TRI + Trail Visual + TRI + Trail Visual + TRI + Trail	Forest + TRI + Roads TRI + Buildings TRI + Hum_passage Roads Forest Buildings
M_h	7153.69	3.96	Roe Red Wolf Roe/Red Roe/Wolf Red/Wolf	Visual + TRI + Trail Visual + TRI + Trail Visual + TRI + Trail	Forest + TRI + Roads Forest + TRI + Buildings TRI + Hum_passage Open + Roads Forest + Hum_passage Forest + Buildings
M_i	7154.89	5.16	Roe Red Wolf Roe/Red Roe/Wolf Red/Wolf	Visual + TRI + Trail Visual + TRI + Trail Visual + TRI + Trail	Forest + TRI + Roads Forest + TRI + Buildings TRI + Hum_passage Open + Buildings Forest + Buildings Forest + Buildings
M_g	7157.69	7.96	Roe Red Wolf Roe/Red Roe/Wolf Red/Wolf	Visual + TRI + Trail Visual + TRI + Trail Visual + TRI + Trail	Forest + TRI + Roads Forest + TRI + Buildings TRI + Hum_passage TRI + Roads Forest + Buildings TRI + Buildings

*Note:* The top model selected for the main analysis is shown in first position for reference, given its lower AIC value. Roe = roe deer, Red = red deer, Wolf = wolf, Visual = visual clarity, Trail = CT positioned on/off a trail, forest/open = % of forest cover/open habitats, TRI = Terrain Ruggedness Index, Roads/Buildings = distance of the CT from the closest road/building, Hum_passage = human capture rate for each CT.

### Detailed Results of Tested Multi‐Species Occupancy Models (MSOMs)

Roe = roe deer, Red = red deer, Wolf = wolf, Visual = visual clarity, Trail = CT on/off a trail, forest/open = % of forest cover/open habitats, TRI = Terrain Ruggedness Index, Roads/buildings = distance of the camera from the closest road/building, Hum_passage = human capture rate for each camera, Human = record of the presence or absence of humans each day, Hunting = record of the days when hunting was active.

(i) Species co‐occurrence.

**TABLE A7:** MSOMs results, evaluating: Independent occurrence of the three species (M1), constant pairwise dependence (M2) and pairwise dependence modelled with covariates (M3).

Species	Covariate	M1			M2			M3		
		Estimate	SE	*p*	Estimate	SE	*p*	Estimate	SE	*p*
Roe deer	Intercept *p* Visual Trail TRI	−1.530 0.413 −0.339 −0.591	0.039 0.044 0.038 0.042	< 0.001* < 0.001* < 0.001* < 0.001*	−1.530 0.413 −0.339 −0.591	0.039 0.044 0.038 0.042	< 0.001* < 0.001* < 0.001* < 0.001*	−1.530 0.413 −0.339 −0.591	0.039 0.044 0.038 0.042	< 0.001* < 0.001* < 0.001* < 0.001*
	*Intercept* ψ forest TRI Roads	5.713 0.110 −4.192 1.879	2.299 0.768 1.961 1.051	0.013* 0.887 0.033* 0.074	5.149 0.004 −4.270 1.802	2.311 0.779 1.937 1.030	0.026* 0.996 0.028* 0.080	6.292 0.845 −5.308 2.006	2.750 0.960 2.234 1.217	0.022* 0.379 0.018* 0.099
Red deer	*Intercept p* Visual Trail TRI	−3.426 0.092 −1.068 0.179	0.141 0.115 0.170 0.116	< 0.001* 0.422 < 0.001* 0.124	−3.426 0.095 −1.068 0.179	0.140 0.115 0.168 0.116	< 0.001* 0.409 < 0.001* 0.124	−3.434 0.098 −1.080 0.184	0.140 0.115 0.168 0.116	< 0.001* 0.391 < 0.001* 0.114
	*Intercept* ψ TRI Buildings	−0.560 −0.256 −1.608	0.415 0.329 0.628	0.177 0.436 0.011*	−1.533 −0.246 −1.597	1.357 0.394 0.630	0.258 0.533 0.011*	−0.408 −1.971 −2.671	1.798 0.797 1.422	0.821 0.013* 0.060
Wolf	*Intercept p* Visual Trail TRI	−4.091 0.243 0.490 0.106	0.1892 0.237 0.121 0.151	< 0.001* 0.305 < 0.001* 0.481	−4.083 0.270 0.476 0.121	0.189 0.238 0.120 0.148	< 0.001* 0.257 < 0.001* 0.415	−4.076 0.276 0.470 0.117	0.188 0.237 0.120 0.147	< 0.001* 0.244 < 0.001* 0.427
	*Intercept* ψ TRI Hum_passage	0.821 0.739 4.232	0.678 0.430 2.244	0.226 0.086 0.059*	−0.432 0.839 4.335	1.327 0.457 2.405	0.745 0.066 0.072	−1.436 1.486 5.744	1.508 0.614 2.860	0.341 0.015* 0.045*
Roe ‐Red	*Intercept* ψ Open Roads				0.627	1.398	0.654	−1.742 −1.290 1.058	1.863 0.593 0.634	0.350 0.030* 0.095
Roe ‐ Wolf	*Intercept* ψ Forest Buildings				0.950	1.354	0.483	1.647 −0.476 0.897	1.502 0.530 0.546	0.273 0.369 0.101
Red ‐ Wolf	*Intercept* ψ Buildings				0.833	0.725	0.250	2.235 −0.198	1.308 1.494	0.088 0.894

*Note:* Statistically significant results are highlighted with an asterisk (*) at *p* < 0.05, and the best model of the set is highlighted in grey.

(ii) The impact of human presence

**TABLE A8:** MSOMs results, with species detection probability modelled as a function of the latent presence/absence of humans, evaluating: independent occurrence of the three species (M4), constant pairwise dependence (M5) and pairwise dependence modelled with covariates (M6).

Species	Covariate	M4			M5			M6		
		Estimate	SE	*p*	Estimate	SE	*p*	Estimate	SE	*p*
Roe deer	Intercept *p* Visual Trail TRI Human	−1.527 0.401 −0.366 −0.569 0.074	0.039 0.044 0.040 0.043 0.033	< 0.001* < 0.001* < 0.001* < 0.001* 0.025*	−1.527 0.401 −0.366 −0.569 0.074	0.039 0.044 0.040 0.043 0.033	< 0.001* < 0.001* < 0.001* < 0.001* 0.025*	−1.527 0.401 −0.366 −0.569 0.074	0.039 0.044 0.040 0.043 0.033	< 0.001* < 0.001* < 0.001* < 0.001* 0.025*
	Intercept ψ Forest TRI Roads	5.714 0.110 −4.193 1.879	2.300 0.768 1.962 1.051	0.013* 0.886 0.033* 0.074	5.155 0.001 −4.275 1.801	2.315 0.779 1.940 1.030	0.026* 0.999 0.028* 0.080	6.294 0.844 −5.309 2.005	2.753 0.962 2.236 1.218	0.022* 0.380 0.018* 0.100
Red deer	Intercept *p* Visual Trail TRI Human	−3.422 0.090 −1.073 0.186 0.056	0.141 0.116 0.170 0.118 0.127	< 0.001* 0.438 < 0.001* 0.113 0.659	−3.422 0.092 −1.073 0.186 0.052	0.140 0.115 0.168 0.118 0.127	< 0.001* 0.423 < 0.001* 0.115 0.683	−3.431 0.096 −1.084 0.190 0.044	0.140 0.115 0.169 0.118 0.127	< 0.001* 0.403 < 0.001* 0.107 0.730
	Intercept ψ TRI Buildings	−0.564 −0.255 −1.614	0.415 0.329 0.630	0.174 0.438 0.010*	−1.534 −0.244 −1.605	1.356 0.394 0.632	0.258 0.535 0.011*	−0.403 −1.969 −2.668	1.799 0.798 1.425	0.823 0.014* 0.061
Wolf	Intercept *p* Visual Trail TRI Human	−4.105 0.226 0.461 0.116 0.062	0.191 0.239 0.128 0.151 0.086	< 0.001* 0.345 < 0.001* 0.442 0.468	−4.099 0.254 0.446 0.131 0.063	0.191 0.240 0.128 0.148 0.086	< 0.001* 0.290 < 0.001* 0.378 0.466	−4.093 0.261 0.440 0.127 0.062	0.190 0.239 0.128 0.147 0.086	< 0.001* 0.274 < 0.001* 0.398 0.469
	Intercept ψ TRI Hum_passage	0.820 0.738 4.205	0.675 0.431 2.235	0.225 0.087 0.060	−0.433 0.836 4.300	1.327 0.458 2.395	0.744 0.068 0.073	−1.433 1.482 5.713	1.510 0.616 2.854	0.342 0.016* 0.045*
Roe ‐Red	Intercept ψ Open Roads				0.622	1.397	0.656	−1.748 −1.284 1.062	1.864 0.593 0.636	0.348 0.031* 0.095
Roe ‐ Wolf	Intercept ψ Forest Buildings				0.950	1.355	0.484	1.648 −0.473 0.898	1.506 0.531 0.551	0.274 0.373 0.103
Red ‐ Wolf	Intercept ψ Buildings				0.832	0.727	0.253	2.219 −0.215	1.313 1.500	0.091 0.886

*Note:* Statistically significant results are highlighted with an asterisk (*) at *p* < 0.05, and the best model of the set is highlighted in grey.

(iii) The effects of hunting

**TABLE A9:** MSOM results, with species detection probability modeled as a function of the hunting period, evaluating: independent occurrence of the three species (M7), constant pairwise dependence (M8) and pairwise dependence modeled with covariates (M9).

Species	Covariate	M7			M8			M9		
		Estimate	SE	*p*	Estimate	SE	*p*	Estimate	SE	*p*
Roe deer	Intercept *p* Visual Trail TRI Hunting	−1.568 0.410 −0.339 −0.610 −0.151	0.040 0.044 0.038 0.042 0.040	< 0.001* < 0.001* < 0.001* < 0.001* < 0.001*	−1.568 0.410 −0.339 −0.610 −0.151	0.040 0.044 0.038 0.042 0.040	< 0.001* < 0.001* < 0.001* < 0.001* < 0.001*	−1.568 0.410 −0.339 −0.610 −0.151	0.040 0.044 0.038 0.042 0.040	< 0.001* < 0.001* < 0.001* < 0.001* < 0.001*
	Intercept ψ Forest TRI Roads	5.710 0.111 −4.190 1.878	2.297 0.768 1.959 1.051	0.013 0.885 0.033* 0.074	5.154 0.003 −4.273 1.803	2.314 0.779 1.939 1.031	0.026* 0.997 0.028* 0.080	6.294 0.837 −5.301 2.000	2.748 0.958 2.231 1.215	0.022* 0.382 0.018* 0.100
Red deer	Intercept *p* Visual Trail TRI Hunting	−3.438 0.094 −1.067 0.174 −0.038	0.149 0.115 0.170 0.117 0.137	< 0.001* 0.415 < 0.001* 0.137 0.782	−3.438 0.097 −1.067 0.174 −0.038	0.147 0.115 0.169 0.118 0.137	< 0.001* 0.402 < 0.001* 0.138 0.780	−3.445 0.100 −1.078 0.179 −0.034	0.148 0.115 0.169 0.118 0.137	< 0.001* 0.385 < 0.001* 0.127 0.803
	Intercept ψ TRI Buildings	−0.559 −0.256 −1.609	0.415 0.329 0.629	0.178 0.436 0.011*	−1.536 −0.246 −1.600	1.357 0.394 0.631	0.258 0.533 0.011*	−0.407 −1.970 −2.682	1.799 0.796 1.428	0.821 0.013* 0.060
Wolf	Intercept *p* Visual Trail TRI Hunting	−4.067 0.240 0.488 0.118 0.090	0.193 0.237 0.121 0.152 0.154	< 0.001* 0.312 < 0.001* 0.439 0.560	−4.058 0.265 0.475 0.132 0.095	0.192 0.238 0.119 0.149 0.154	< 0.001* 0.266 < 0.001* 0.375 0.536	−4.050 0.270 0.470 0.128 0.097	0.190 0.237 0.119 0.148 0.154	< 0.001* 0.255 < 0.001* 0.385 0.532
	Intercept ψ TRI Hum_passage	0.808 0.733 4.207	0.673 0.428 2.228	0.230 0.087 0.059*	−0.445 0.836 4.316	1.321 0.457 2.395	0.736 0.067 0.072	−1.438 1.480 5.735	1.502 0.612 2.853	0.338 0.016* 0.044*
Roe ‐Red	Intercept ψ Open Roads				0.627	1.398	0.654	−1.747 −1.289 1.059	1.861 0.592 0.634	0.348 0.030* 0.095
Roe ‐ Wolf	Intercept ψ Forest Buildings				0.949	1.349	0.482	1.640 −0.474 0.893	1.497 0.529 0.543	0.273 0.370 0.100
Red ‐ Wolf	Intercept ψ Buildings				0.841	0.724	0.245	2.243 −0.182	1.309 1.498	0.087 0.904

*Note:* Statistically significant results are highlighted with an asterisk (*) at *p* < 0.05, and the best model of the set is highlighted in grey.

### Single‐Species Versus Multi‐Species Occupancy Models

This section compares the effect of single‐species and multi‐species occupancy models on the estimated marginal occupancy values for each species. The human‐related variable used in each species' individual occupancy model is shown in the plots (roe deer: distance from roads, red deer: distance from buildings, wolf: human passage rate) (Figure A1).
